# Supplementary material for: Dynamic effects of irrigation on photosynthesis and yield-related physiological characteristics in different glutinous wheat cultivars
Source: PeerJ. 2025 Oct 24;13:e20230. doi: 10.7717/peerj.20230 (PMC12558156; doi:10.7717/peerj.20230)
Supplement: Supplemental Information 10 [file peerj-13-20230-s010.docx]

Translations and Explanations for Non-English Content in Fig2to3May_6th

| Original Text | Translation | Note |
| --- | --- | --- |
| A470结果 | A470 Value | POD activity, calculated from four time-point A470 readings |
